# The impact of structural integrity and route of administration on the antibody specificity against three cow’s milk allergens - a study in Brown Norway rats

**DOI:** 10.1186/2045-7022-4-25

**Published:** 2014-08-18

**Authors:** Jeanette Lund Madsen, Stine Kroghsbo, Charlotte Bernhard Madsen, Irina Pozdnyakova, Vibeke Barkholt, Katrine Lindholm Bøgh

**Affiliations:** 1 Division of Toxicology and Risk Assessment, National Food Institute, Technical University of Denmark, Mørkhøj Bygade 19, DK-2860 Søborg, Denmark; 2 NNF Center for Protein Research, University of Copenhagen, Copenhagen, Denmark; 3 Department of Systems Biology, Enzyme and Protein Chemistry, Technical University of Denmark, Kongens Lyngby, Denmark

**Keywords:** Food allergy, Cow’s milk allergens, IgE, Conformational epitopes, Linear epitopes, Animal model, Oral administration, i.p. immunisation

## Abstract

**Background:**

Characterisation of the specific antibody response, including the epitope binding pattern, is an essential task for understanding the molecular mechanisms of food allergy. Examination of antibody formation in a controlled environment requires animal models. The purpose of this study was to examine the amount and types of antibodies raised against three cow’s milk allergens; *β*-lactoglobulin (BLG), *α*-lactalbumin (ALA) and *β*-casein upon oral or intraperitoneal (i.p.) administration. A special focus was given to the relative amount of antibodies raised against linear versus conformational epitopes.

**Methods:**

Specific antibodies were raised in Brown Norway (BN) rats. BN rats were dosed either (1) i.p. with the purified native cow’s milk allergens or (2) orally with skimmed milk powder (SMP) alone or together with gluten, without the use of adjuvants. The allergens were denatured by reduction and alkylation, resulting in unfolding of the primary structure and a consequential loss of conformational epitopes. The specific IgG1 and IgE responses were analysed against both the native and denatured form of the three cow’s milk allergens, thus allowing examination of the relative amount of linear versus conformational epitopes.

**Results:**

The inherent capacity to induce specific IgG1 and IgE antibodies were rather similar upon i.p. administration for the three cow’s milk allergens, with BLG = ALA > *β*-casein. Larger differences were found between the allergens upon oral administration, with BLG > ALA > *β*-casein. Co-administration of SMP and gluten had a great impact on the specific antibody response, resulting in a significant reduced amount of antibodies. Together results indicated that most antibodies were raised against conformational epitopes irrespectively of the administration route, though the relative proportions between linear and conformational epitopes differed remarkably between the allergens.

**Conclusions:**

This study showed that the three-dimensional (3D) structure has a significant impact on the antibodies raised for both systemic and orally administered allergens. A remarkable difference in the antibody binding patterns against linear and conformational epitope was seen between the allergens, indicating that the structural characteristics of proteins may heavily affect the induced antibody response.

## Introduction

Food allergy is a major health problem of growing concern in Western countries [1-4]. The mechanisms underlying IgE-mediated allergy are only poorly resolved. An understanding of the basic molecular mechanisms involved in the induction of food allergy is based on the knowledge about the antibody binding sites on the allergens, the epitopes. Understanding the nature of IgE inducing epitopes will elucidate how allergenic potential relates to protein structure. This is likewise the “breeding ground” for development of new diagnostic, prophylactic as well as therapeutic strategies for food allergy. Antibody epitopes are divided into linear and conformational epitopes, based on the proximity of the amino acids in the primary sequence of the protein that are involved in antibody binding. Epitopes constituted of a continuous stretch of amino acids are designated linear epitopes, while epitopes constituted of amino acids distant from each other in the primary structure but brought together by the secondary, tertiary or quaternary folding of the protein are designated conformational epitopes [5,6]. Antibody binding epitopes are generally thought to be conformational in nature [7-10]. Yet for food allergens, by far most linear epitopes have been identified and a particular importance for this type of epitopes has been suggested in the IgE-mediated food allergic disease [11].

The three-dimensional (3D) structure defines the amino acid residues on the surface of an allergen available for antibody binding. Thus the accessibility of a given epitope may greatly be influenced by the structural folding of the allergen. Food allergens possess different protein chemical properties and thus differ in their structural stability. The microenvironment, such as pH, ionic strength, binding to other molecules and/or the state of breakdown, may affect this stability, thus to various degree depending on the allergen. Such environmental changes take place during the digestive process in the gastrointestinal (GI) tract and greatly affect the folding of the allergen and hence the shape in which the allergen is presented to the immune system. Thereby the passage through the GI tract may have a huge impact on the number and type of epitopes accessible for antibody binding.

In order to study formation of antibodies directed against food allergens in a controlled environment, animal models are needed. Various animal models for food allergy exist, differing in the choice of administration route, use of adjuvant and whether the allergen is administered alone or as part of a whole food. The impact of such choices on the specificities of the antibodies raised is not fully resolved.

Cow’s milk allergy (CMA) is the most common type of IgE-mediated food allergy in young children, affecting around 2.5% of children below the age of 3 years [12,13]. Cow’s milk contains around 20 proteins able to induce IgE-mediated allergy [14], of which 10 are reported in the official Allergen Nomenclature Database from the World Health Organization and International Union of Immunological Societies (WHO/IUIS) (http://www.allergen.org). *β*-lactoglobulin (BLG) officially designated Bos d 5, *α*-lactalbumin (ALA) officially designated Bos d 4 and caseins, officially designated Bos d 8, are the most important and major cow’s milk allergens [15]. These allergens possess different protein chemical features, some of which may influence their allergy-inducing capacity in quantity as well as quality, such as structural stability to processing, including digestion [15-18].

The aim of this study was to investigate the relative importance of linear versus conformational antibody epitopes of the three cow’s milk allergens; BLG, ALA and *β*-casein. Denaturation of proteins will result in the unfolding of the native allergen structure. Thus the linear epitopes are maintained but there is a consequential loss of conformational epitopes [16]. Hence the relative ratio of linear and conformational antibody binding epitopes can be identified by measuring the decrease (or increase) in the antibody binding upon loss of the native 3D structure due to the denaturation process [6]. We have taken the advantages of this approach to compare the binding capacity against native and denatured BLG, ALA and *β*-casein of IgG1 and IgE antibodies raised in Brown Norway (BN) rats dosed either i.p. or orally. Further we aimed to study the influence of co-administration with other proteins on the amount of antibody raised in the BN rats.

## Materials and methods

### Allergens

BLG was from a pilot batch purified and kindly delivered by Arla Food Ingredients (Videbæk, Denmark), ALA (61289, Fluka, Sigma, St. Louis, MO, USA) and *β*-casein (C6905, Sigma) was from Sigma. The protein content and characteristics of these three cow’s milk proteins are summarised in Table 1. Skimmed milk powder (SMP) (70166 Fluka, Sigma) and wheat gluten (G5004, Sigma) were likewise from Sigma. The protein content of BLG, ALA and *β*-casein in SMP is equivalent to cow’s milk (Table 1).

**Table 1:** **Characteristics of the three cow’s milk allergens: BLG, ALA and *β*-casein**

**Source**	**Allergen**^**a**^	**Amount in cow’s milk (%)**^**b**^	**Size (kDa)**^**a,b**^	**Cysteins**^**b**^	**Disulfide bonds**^**b**^	**Stability to digestion**^**c**^	**Importance of the allergen**^**a,b**^
Whey	BLG (Bos d 5)	10	18.3	5	2	Yes	Major
	ALA (Bos d 4)	5	14.2	8	4	No	Major
Caseins	*β*-casein (Bos d 11)	28	23.6	0	0	No	Major

^a^http://www.allergen.org, [19], ^b^[15], ^c^[18].

### Denaturation of allergens

Samples of the three individual cow’s milk allergens; BLG, ALA and *β*-casein as well as samples of digoxigenin-coupled BLG and ALA were concentrated by evaporation in SpeedVac. A solution of 6 M guanidine-HCl (G4505, Sigma), 0.5 M Tris-HCl (A1087.1000, AppliChem, Darmstadt, Germany) and 0.01 M. The EDTA concentration is 0.01 M (1.08418, Merck, Darmstadt, Germany) pH 8.6 was added to the allergens to obtain an allergen concentration of 5 mg/mL. Subsequently dithiothreitol (DTT, D0632, Sigma) was added to a final concentration of 0.1 M and the solutions were saturated with argon, capped and incubated for 2 h at 50°C. Iodoacetamide (I1149, Sigma) diluted in 0.5 M Tris-HCl, pH 8.6 was added to the solution to give a final concentration of 0.24 M. After incubation for 30 min at room temperature (RT) 2-mercaptoethanol (M7522, Sigma) was added to give a concentration of 2.4 M. Lastly, the solutions were placed in dialysis-tubes with a pore size of 6-8 kDa (Spectra/Por®Dialysis Membrane MWCO: 6-8000, Spectrum Laboratories, Inc., Rancho Domingues, CA, USA), and dialysed against PBS (137 mM NaCl, 3 mM KCl, 8 mM Na_2_HPO_4_, 1 mM KH_2_PO_4_, pH 7.2) for three days at 4°C and afterwards stored at -20°C until further use.

### Native PAGE

To test the degree of modification of BLG, ALA and *β*-casein, a native gel electrophoresis was performed using a Criterion TGX Tris-HCl Leammli-like 10-20% gel (567-1113, Bio-Rad, Hercules, CA, USA). Native and denatured samples of the three allergens (~2-5 μg), diluted in native sample buffer (161-0738, Bio-Rad) were loaded into the gel. Gel electrophoresis was performed in Tris/Glycine running buffer (161-0734, Bio-Rad) diluted 1:10 (v:v) in double distilled water for 55 min at 200V constant current at RT. Staining was performed with Bio-Safe™ Coomassie (161-0786, Bio-Rad) for 1 h at RT. The gel was photographed using Universal Hood Gel-imager (Bio-Rad, Segrate, Milan, Italy) and the program Quality One.

### Circular dichroism (CD)

Far-UV CD spectra (200-250 nm) were recorded at 20°C in a 1 mm quartz cuvette on a JASCO J815 CD spectrometer (Jasco corporation, Tokyo, Japan) equipped with Peltier thermostatted cell holder. Protein samples were prepared in PBS (137 mM NaCl, 3 mM KCl, 8 mM Na_2_HPO4, 1 mM KH_2_PO4, pH 7.2). Final protein concentrations were 0.5 mg/ml for ALA and BLG, and 0.25 mg/ml for *β*-casein. Spectra (10 accumulations for each sample) were acquired at scan rate of 50 nm/min with a response time of 2 seconds. For each sample 10 scans were recorded and averaged. Buffer (PBS) spectrum was recorded under the same conditions and subtracted from the sample spectra. Protein concentration in each CD sample was determined by UV absorbance at 280 nm. Extinction coefficients at 280 nm were calculated based on amino acid sequence using ProtParam online tool [20]. CD signal is reported as mean residue molar ellipticity (deg*cm^2^*dmol^-1^).

### Animals

At an age of three weeks, BN rats from our in-house breeding colony (National Food Institute, Technical University of Denmark, Denmark) were weaned. They were housed in macrolon cages (2-3 per cage), with a 12 hour light:dark cycle, at a temperature of 22 ± 1°C and a relative humidity of 55 ± 5%. Rats were observed twice daily and clinical signs were recorded. Rats were kept on a diet free of milk and wheat for at least three generation to avoid tolerance to the studied allergens. Diet and acidified water (pH 3.5) were given *ad libitum*. Animal experiments were carried out at the National Food Institute facilities. Ethical approval was given by the Danish Animal Experiments Inspectorate and the authorisation number given 2009/561-1710. The experiments were overseen by the National Food Institutes in-house Animal Welfare Committee for animal care and use.

### I.p. study with purified native BLG, ALA and *β*-casein

**Figure 1:**
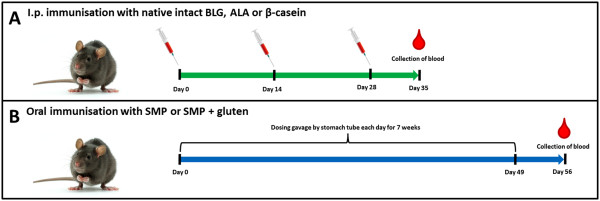
**Overview of the animal experimental designs. (A)** BN rats, 12 per group, were immunised i.p. with purified native intact BLG, ALA or *β*-casein three times day 0, 14 and 28 without the use of adjuvant. Rats were sacrificed one week after last immunisation at day 35. **(B)** BN rats, 10 per group, were immunised by gavage each day for seven weeks with SMP alone or SMP together with wheat gluten without the use of adjuvant. Rats were sacrificed one week after last immunisation at day 56.

To study the specific antibody response raised against the native purified intact allergens, BN rats 4-9 weeks of age were allocated into three groups of 12 rats (n = 6 per sex). Rats were immunised i.p. three times with 200 μg of purified intact BLG, ALA or *β*-casein in PBS at day 0, 14 and 28. One week after the last immunisation rats were sacrificed and blood was collected. See Figure 1A for an overview of the animal experimental design.

### Feeding study with SMP

For examination of the specific antibody response against BLG, ALA and *β*-casein as part of a whole food and by means of the oral route, BN rats were dosed by gavage with SMP. To study the influence of the presence of additional proteins another group of BN rats were dosed by gavage with SMP together with wheat gluten. Female rats 6-10 weeks of age were allocated into two groups of 10 rats and dosed by gavage each day for 49 days with 0.5 mL per rat per day of either SMP (~60 mg protein) alone suspended in corn oil or with SMP (~60 mg protein) together with wheat gluten (~20 mg) suspended in corn oil. One week after the last dosing rats were sacrificed and blood collected. See Figure 1B for an overview of the animal experimental design.

### Indirect ELISA for detection of BLG, ALA and *β*-casein specific IgG1

In order to test the specific IgG1 response to native as well as denatured BLG, ALA and *β*-casein, indirect ELISAs were performed. Maxisorp microtitre plates (96-well, Nunc, Roskilde, Denmark) were coated with 100 μL/well of 10 μg/mL of allergen diluted in coating buffer (15 mM Na_2_CO_3_, 35 mM NaHCO_3_, pH 9.6) and incubated overnight at 4°C. Plates were washed five times between each step in PBS with 0.01% Tween 20 (PBS-T). For *β*-casein only, plates were blocked with 200 μL/well of 1% ovalbumin (A-5503, Sigma) in PBS-T (w:v) for 1 h at 37°C. Two-fold serial dilutions of serum (starting at 1:8, v/v) in PBS-T, 50 μL/well, were added and incubated for 1 h at RT. For detection, 50 μL of HRP-labelled mouse-α-rat IgG1 (3060-05, Southern Biotech, Birmingham, AL, USA) diluted 1:20,000 (v:v) in PBS-T was added to each well and incubated for 1 h at RT. For visualisation of the specific antibody response, 100 μL/well of 3,3’,5,5-tetramethylbenzidine (TMB)-one (4380A, Kem-En-Tec Diagnostic, Tåstrup, Denmark) was added to each well and incubated at RT for approximately 12 min, where reaction was stopped by adding 100 μL/well of 0.2 M H_2_SO_4_. Absorbance was measured at 450 nm with a background reference at 630 nm using a microtitre reader (EL 800, BioTek, Winooski, VT, USA). Data are expressed as Log_2_ titre values and defined as the interpolated dilution of the given serum sample leading to the mean absorbance for the negative control serum sample +3 SD, corresponding to cut off values of 0.1 for BLG and *β*-casein and 0.15 for ALA.

### Antibody-capture ELISA for detection of BLG, ALA and *β*-casein-specific IgE

In order to test the specific IgE responses to native BLG, ALA and *β*-casein and denatured BLG and ALA, antibody-capture ELISAs were performed. The amount of specific IgE antibodies only account for a small fraction of the total sum of specific antibodies, for which reason an antibody-capture ELISA is a far more sensitive choice than an indirect ELISA for most allergens. The ELISAs were performed with native or denatured digoxigenin-coupled BLG, native or denatured digoxigenin-coupled ALA and digoxigenin-coupled *β*-casein essentially as described in Bøgh *et al.*[21]. ELISAs were not performed with denatured digoxigenin-coupled *β*-casein due to shortness of the *β*-casein source. Data are expressed as Log_2_ titre values and defined as the interpolated dilution of the given serum sample leading to the mean absorbance for the negative control serum sample +3 SD, correlating to cut off values of 0.4 for native BLG, 0.2 for native and denatured ALA and *β*-casein and 0.15 for denatured BLG.

### Statistical analysis

ELISA results expressed as Log_2_ antibody titres were examined for group differences, using the non-parametric one-way ANOVA, Kruskal-Wallis test, followed by Dunn’s multiple comparison of three or more groups. For comparison of two groups the non-parametric Mann-Whitney test was used. Differences between groups of animals were regarded as significant when *P* ≤ 0.05. Asterisks indicate a statistically significant difference between two given groups: * = *P* ≤ 0.05, ** = *P* ≤ 0.01, and *** = *P* ≤ 0.001

## Results

### Immunogenic and sensitising potency of BLG, ALA and *β*-casein

**Figure 2:**
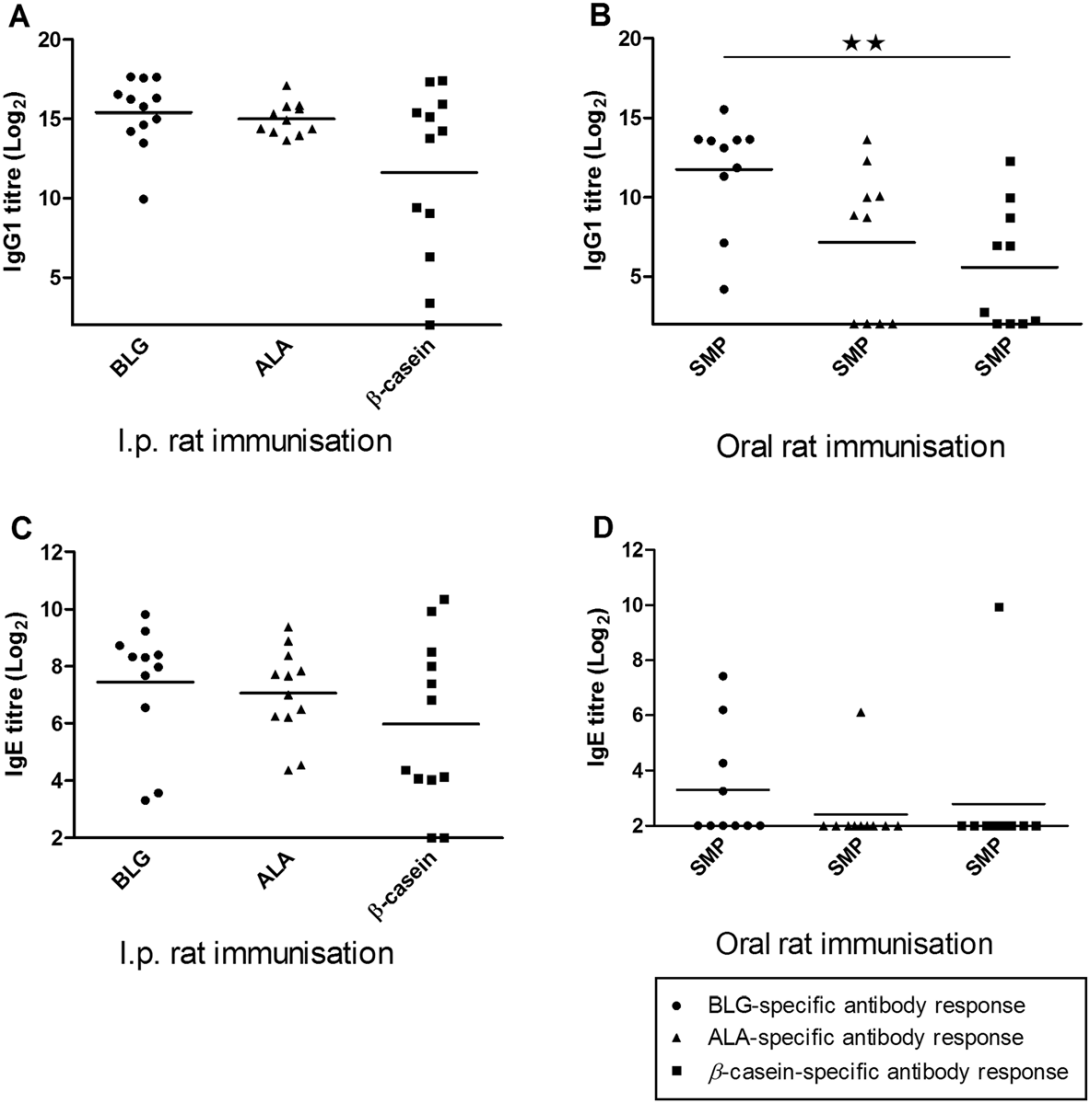
**Immunogenicity and sensitising capacity of the cow’s milk proteins.** The titre levels of the specific antibodies raised in rats dosed i.p. or orally with cow’s milk proteins. **(A)** BLG (●), ALA (■) or *β*-casein (▲)-specific IgG1 responses for rats immunised i.p. three times at an interval of 14 days with either BLG, ALA and *β*-casein, respectively, without the use of adjuvant. **(B)** BLG (●), ALA (■) and *β*-casein (▲)-specific IgG1 responses for rats dosed orally by SMP each day for seven weeks without the use of adjuvant. **(C)** BLG (●), ALA (■) and *β*-casein (▲)-specific IgE responses for rats immunised i.p. three times at an interval of 14 days with either BLG, ALA or *β*-casein without the use of adjuvant. **(D)** BLG (●), ALA (■) and *β*-casein (▲)-specific IgE responses for rats dosed orally by SMP each day for seven weeks without the use of adjuvant.

The cow’s milk allergen-specific IgG1 and IgE responses raised in rats administered either i.p. with the purified native intact allergens or orally by gavage with SMP were evaluated by means of ELISAs. Results indicated that the inherent immunogenicity as well as sensitising capacity differed only slightly between the three cow’s milk allergens (Figure 2 A and C). BLG and ALA were shown to induce a similar but somewhat higher IgG1 and IgE antibody response than *β*-casein when administered i.p. in identical amounts (BLG = ALA > *β*-casein). Furthermore the *β*-casein differed from BLG and ALA by showing a much greater variation in the specific IgG1 response between individual rats. Administration of allergens by the oral route resulted in specific antibody titre levels which differed more between the three cow’s milk allergens than did the i.p. administration. The highest IgG1 response was found for BLG followed by ALA and *β*-casein (BLG > ALA > *β*-casein). The immunogenicity of ALA was reduced compared to BLG when the administration route was changed from i.p. to oral (Figure 2 A and B). This variation in the induced immunogenicity cannot solely be a result of the various amount of the three cow’s milk allergens present in SMP, as *β*-casein are the one present in largest amount but still the one showing the lowest immunogenicity. The sensitising capacity of the three cow’s milk allergens after oral administration was reduced compared to the sensitising capacity obtained after i.p. immunisation, with only one measurable IgE responder for both ALA and *β*-casein, and four for BLG (Figure 2 D). Overall these results indicate that the choice of administration route may heavily influence the amount of specific antibodies raised and thereby the interpretation of the difference in immunogenicity as well as sensitising potency between various allergens. Hence, the site of presentation to the immune system as well as the digestion in the GI tract may greatly affect the quantity of specific antibodies induced.

### The impact of immunising SMP together with gluten proteins

**Figure 3:**
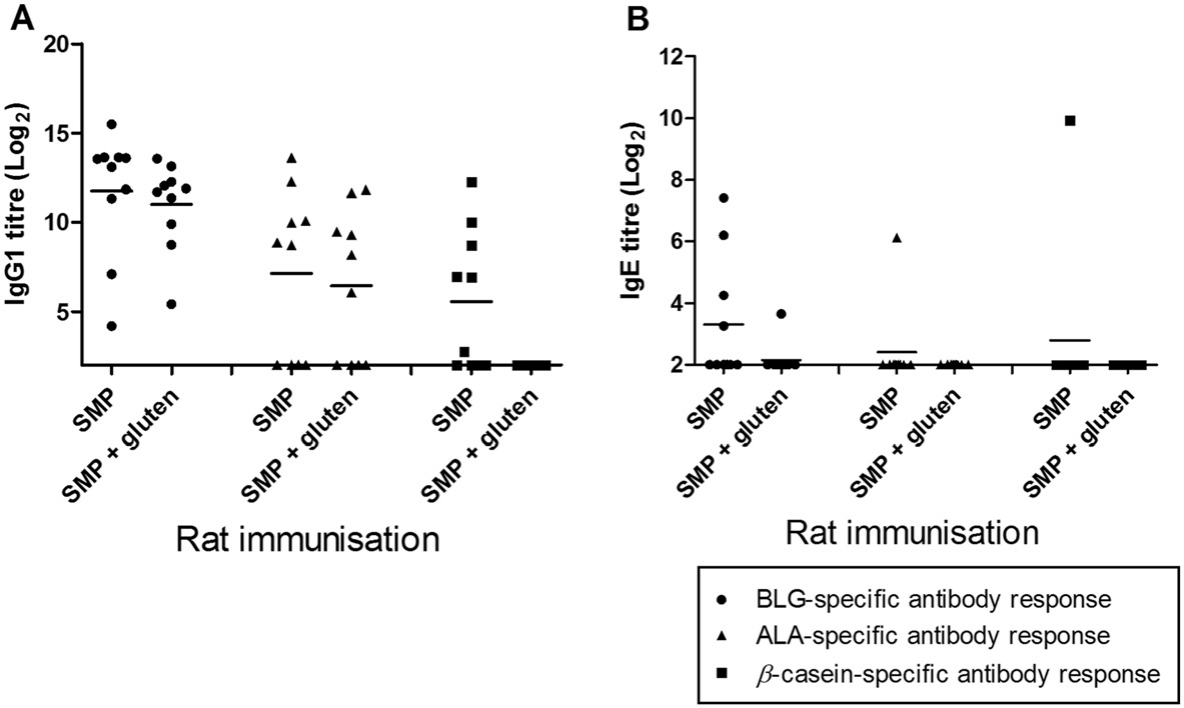
**Impact of co-administration with gluten.** Comparison of the specific antibody responses between rats dosed orally with SMP alone or with SMP together with gluten proteins. **(A)** BLG (●), ALA (■) and *β*-casein (▲)-specific IgG1 responses and **(B)** BLG (●), ALA (■) and *β*-casein (▲)-specific IgE responses.

To study the effect of co-administration with other proteins on the allergen-specific immune response, BN rats were in addition to SMP alone dosed with SMP together with gluten proteins. From Figure 3 it is clearly shown that co-administration of SMP together with gluten protein influences the immunogenicity as well as the sensitising capacity of the three cow’s milk protein. For all three allergens the specific IgG1 response was reduced when SMP was administered together with gluten compared to SMP alone, and for *β*-casein the specific response was completely abrogated. This indicates that the immune response directed against one protein can be affected by other proteins.

### Degree of cow’s milk allergen denaturation/modification

**Figure 4:**
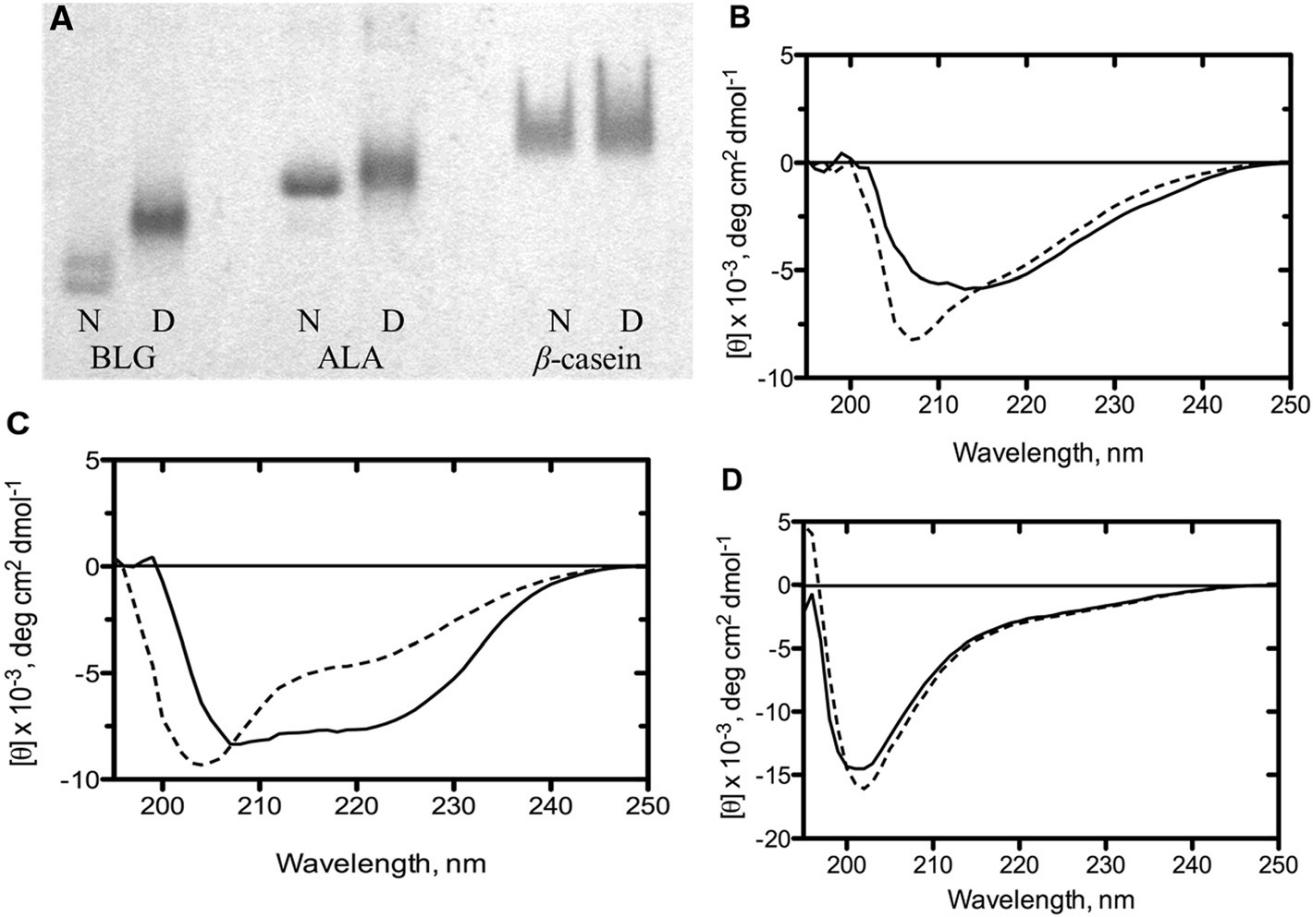
**Protein modifications induced by the denaturation process.** Native PAGE for native (N) and denatured (D) BLG, ALA and *β*-casein **(A)** and CD spectra of BLG **(B)**; ALA **(C)** and *β*-casein **(D)** in native (solid line) and denatured (dashed line) states. All spectra were recorded in PBS buffer at 20°C in a 1-mm quartz cuvette.

Unfolding of the three cow’s milk allergens was performed by reduction and alkylation. The degree of structural modification resulting from the denaturation procedure can be elucidated by electrophoresis in native gels performed without reducing agents, as the protein mobility will largely depend on the protein shape. Though the native PAGE is not an absolute measure of changes in shape, it appears from the native gel that BLG has been subjected to the largest structural changes. BLG emerged as two sharp bands in native form, whereas the denatured BLG emerged as one wider band with reduced motility (Figure 4 A). ALA appeared with similar motility for native and denatured state, though the denatured band was wider and more diffuse. In contrast, the native and denatured form of *β*-casein had very similar appearance in the gel.

For further evaluation of the extent of structural changes achieved by reduction and alkylation, the secondary structure of the native and reduced and alkylated proteins was analysed by far-UV CD (Figure 4 B-D). The native state of BLG exhibits a typical spectrum of a protein with predominantly *β*-sheet structure and a low helical content, which is characterised by a broad minimum around 215 nm and slight shoulder around 210 nm. (Figure 4 B). The spectrum of reduced and alkylated BLG has a distinct shape with a minimum at 207 nm. The observed shift of the peak position from 215 nm to 207 nm indicates an increase in the amount of unfolded conformations. The native ALA produces a classical helical CD spectrum with two broad negative peaks at 222 and 208 nm (Figure 4 C). The reduced and alkylated ALA exhibits a negative peak at 204 nm and a shoulder around 225 nm, which is indicative of largely unfolded protein albeit with some degree of residual secondary structure. In contrast to BLG and ALA, CD spectrum of the native *β*-casein is not significantly different for the spectrum of the reduced and alkylated form of the protein (Figure 4 D). Both forms of *β*-casein show major peak at 204 nm and a shoulder around 230 nm, which are the spectral features of proteins lacking a well-defined secondary structure. The reduce and alkylated *β*-casein shows a slight increase in the depth of the trough at 204 nm as compared to the native *β*-casein, but this subtle difference is difficult to interpret in terms of changes in protein conformation. Together these results clearly demonstrate that especially BLG and ALA have been subjected to large structural modifications from the denaturation process, whereas the extent of structural changes for *β*-casein are difficult to interpret, probably due to the unstructured and flexible folding of the native form of this protein.

### Linear versus conformational epitope recognition patterns of cow’s milk allergens

**Figure 5:**
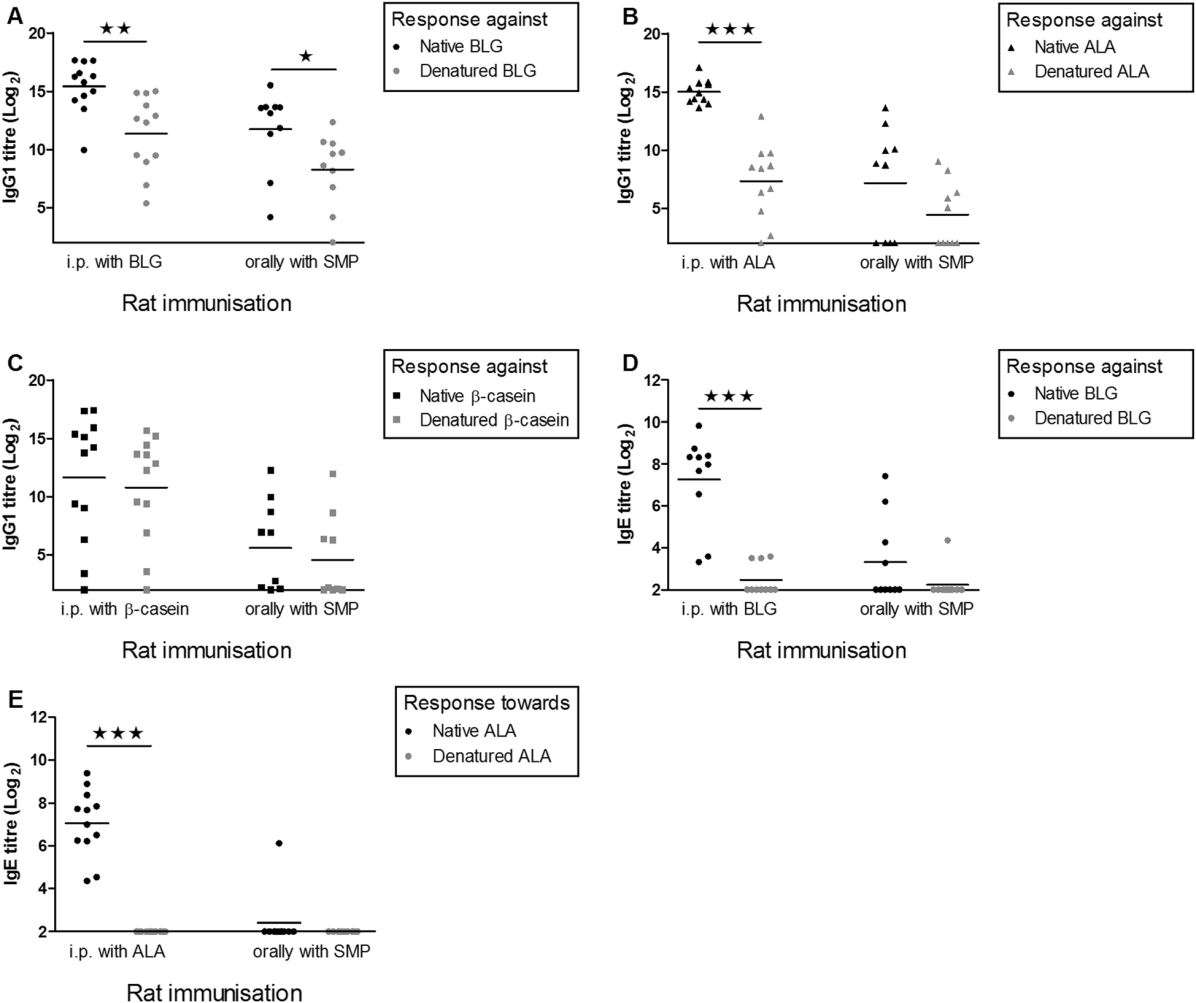
**Comparison of antibody responses against native and denatured cow’s milk proteins.** Sera from BN rats dosed i.p. with purified native intact cow’s milk allergens or orally with SMP were evaluated for the relative importance of linear versus conformational epitopes to which antibodies were raised. The reactivity against native versus denatured proteins of specific IgG1 antibodies raised against **(A)** BLG **(B)** ALA and **(C)***β*-casein or specific IgE antibodies raised against BLG **(D)** and ALA **(E)**.

In order to compare the reactivity of cow’s milk allergen-specific antibodies against linear and conformational epitopes, sera from rats dosed either with the purified native allergens i.p. or with SMP by gavage were analysed for binding reactivity against both the native and denatured allergens in ELISAs. Denaturation of proteins lead to a loss of the 3D folding and thereby a consequential loss of conformational epitopes. Irrespective of the cow’s milk allergen and administration route (i.p. versus oral) a reduction in antibody binding capacity was evident for denatured compared to the native allergens (Figure 5). This shows that for all three cow’s milk allergens, regardless of the administration route, conformational epitopes are of importance. However, the proportion of antibodies reacting with the denatured allergen (i.e. linear epitopes) and antibodies reacting with the native allergen (i.e. linear as well as conformational epitopes) differed remarkably between the three cow’s milk allergens.

For BLG a statistically significant higher IgG1 antibody response could be observed against the native compared to denatured allergen, regardless of rats been dosed i.p. or orally (Figure 5 A). Based on the mean serum dilution factor (2^titre value^) giving a specific response for each group of rats in ELISA, the BLG-specific IgG1 results showed an approximate ratio between linear and conformational epitopes of *1:16* for i.p. and an approximate ratio of *1:12* for oral administration. A similar pattern was seen for specific IgE, where an even higher proportion of the antibodies seemed to be raised against conformational epitopes, as a ratio of *1:23* in linear versus conformational epitopes was indicated after i.p. sensitisation with BLG (Figure 5 D).

For ALA the vast majority of antibodies were raised against conformational epitopes upon administration by i.p. route. ALA-specific IgG1 results showed an approximate ratio of *1:59* for linear vs. conformational epitopes (Figure 5 B), whereas ALA-specific IgE results indicated that IgE antibodies were only raised against conformational epitopes, as no binding to denatured ALA was detected (Figure 5 E). This indicates that ALA primarily induces antibodies against conformational epitopes when presented to the immune system in its native state. In contrast to BLG, a huge reduction in the importance of conformational epitopes were seen when ALA were administered by the oral route and thereby presented to the rat immune system after exposure to the acidic and proteolytic environment in the GI tract. When administered orally, conformational epitopes were still found to be in excess, however the approximate ratio of linear vs. conformational epitopes was now reduced to *1:7* for IgG1. This indicates that for ALA the epitope recognition profile is greatly influenced by the administration route.

In contrast to BLG and ALA, *β*-casein showed a much greater importance of linear epitopes (Figure 5 C). The antibody reactivity against the native and denatured *β*-casein differed only by one titre value, indicating that approximately half of all *β*-casein specific antibodies were raised against conformational epitopes and the other half against linear epitopes. Similarly to BLG, the importance of conformational epitopes seemed not to differ between the two administration routes showing an approximate ratio of linear vs. conformational epitopes of *1:1*.

The proportion of antibodies directed against linear and conformational epitopes in individual rats differed greatly demonstrating the heterogeneity of antibody responses in rats. Whereas all rats, independent on the administration route, raised the greatest amount of antibodies against conformational BLG epitopes compared to linear BLG epitopes, a single rat recognised the denatured ALA better than the native ALA when administered by oral route, and for *β*-casein several rats reacted with the greatest response against the denatured *β*-casein compared to native *β*-casein, demonstrating a greater importance of just linear epitopes compared to conformational epitopes for these rats (data not shown).

## Discussion

When studying food allergens, characterisation of the specific antibody response is important. To understand how allergenic potential relates to the protein structure we have examined the amount and types of antibodies raised against three cow’s milk allergens, with reference to their reactivity to the native and denatured form of the allergen.

Animal models have been used to study the immunogenicity and allergenicity of various food allergens, using various routes of administration with purified proteins or whole foods, with or without the use of adjuvant [22,23]. In this study we used BN rats. The BN rat is a high Ig, particularly IgE-responder strain which to a certain degree resembles atopic humans in their predisposition to develop IgE-mediated allergy [24]. Moreover, it is an animal strain regarded as a useful model for studying sensitisation to food proteins [25,26], since it generates IgG and IgE antibodies of similar protein specificity [24,25] as well as similar epitope specificity [27,28] to those produced in humans. The inherent capacity of the three cow’s milk allergens to induce a specific IgG1 and IgE response following systemic (i.p.) administration showed only minor differences. All three allergens induced high antibody titres, though *β*-casein induced a little lower mean antibody titre than BLG and ALA, due to a large variance in its immunogenicity and sensitising capacity between individual rats. In the context of food allergy, oral administration is the most appropriate route of exposure as this is considered to be the primary route of sensitisation [18,22,29,30]. A greater variation in the immunogenicity between the three cow’s milk allergens was seen upon oral administration compared to i.p. administration. Whereas BLG was able to induce a specific IgG1 response in all rats, only 6 out of 10 rats responded to ALA and 5 out of the 10 rats to *β*-casein after oral administration. This could indicate that the inherent immunogenic capacity of food allergens may be modulated by the modifications resulting from the acidic and proteolytic environment in the GI tract and the way in which they are presented to the immune system. The specific antibody responses upon oral administration correlate well with the susceptibility of the three cow’s milk allergens to digestion. BLG has been demonstrated to be a protein resistant to digestion in contrast to both ALA and *β*-casein that are easily digestible proteins [18], probably resulting in a lower chance for ALA and *β*-casein than BLG to survive the digestion process in the GI tract and survive in a form retaining enough structure and size to be recognised by the immune cells of the inductive immune system. Even fewer rats produced IgE upon oral administration, illustrating the challenge of studying food allergy by oral route without the use of adjuvant.

Foods consist of fats, carbohydrates, proteins and micronutrients, which may all affect the immunogenic and allergenic potential of allergens [31]. There is however no general rule concerning how different proteins are affected by other food components [16]. Co-administration of the cow’s milk proteins together with wheat gluten resulted in a decreased immunogenicity of BLG and ALA and an abrogation of the immunogenicity of *β*-casein by oral administration. This clearly demonstrated that the immunogenicity and allergenicity of a given protein may be affected by the presence of other proteins and that the choice of immunising with a purified protein or with the protein as part of a whole food may greatly influence the resulting outcome and the frequency of animal responders. The immunemodulatory capacity of gluten could be a result of several factors. In a study by Kato *et al*. [32] it was demonstrated that co-administration of ovomucoid with wheat gluten resulted in formation of high-molecular weight complexes leading to aggregation and insolubility of ovomucoid. Binding of the cow’s milk allergens to the wheat gluten proteins could similarly result in a decreased solubility and hence availability of the proteins for interaction with the immune system [33]. A decrease in allergenicity has been shown for BLG after binding to other proteins [34]. Another explanation may be that gluten contains some immunomodulatory properties, as accumulating data suggest that food proteins may act as adjuvant and directly activate the innate immune system [35,36]. This provides the possibility that the gluten proteins contain properties suppressing a Th2 screwed response.

Disruption of the allergen structure by denaturation results in unfolding of the protein and thereby loss of the original structural organisation. As a consequence conformational epitopes will be lost while linear epitopes are still present. Therefore, studying the differences in antibody responses against denatured and native forms of allergens, allows for investigation of the approximate proportion between linear and conformational epitopes. Thus, to evaluate the proportion of antibodies raised against linear versus conformational epitopes of the three cow’s milk allergens by means of i.p. and oral administration a comparison of antibody reactivity against the denatured and native allergens was performed. For all three allergens, irrespectively of the administration route, a reduced (or even eliminated) reactivity to the unfolded allergens compared against the native allergens was revealed. This clearly shows the importance of the 3D structure and thus the very importance of conformational epitopes. Whether these findings can be directly translated into the human situation needs to be determined, but is in line with the recent growing body of evidence supporting a significant role from conformational epitopes in food allergy [18,28,37-46]. The relative importance of linear and conformational epitopes differed remarkably between the three cow’s milk allergens. From the i.p. immunisation study it was demonstrated that antibodies raised against native ALA had the greatest dependency of the 3D structure, followed by BLG. In contrast, *β*-casein raised antibodies reactive to linear and conformational epitopes, in approximately equal amounts. The enormous difference in binding pattern to epitope type between the three allergens could reflect their differences in structure, where both BLG and ALA are small structurally stable and compact proteins possessing two and four disulfide bonds, in contrast to *β*-casein which is regarded an unstructured and flexible protein [15].

Dosing animals by the oral route, the relative amount of linear versus conformational epitope was shown to be the same as for i.p. immunisation for BLG as well as for *β*-casein. In contrast, the amount of conformational epitopes was strongly reduced for ALA upon oral administration compared to systemic administration. The study showed that i.p. immunisation for two of the cow’s milk allergens seemed to be representative for oral dosing, while for one allergen the types of epitopes differed according to administration route.

The GI tract environment may alter the protein structure in a way which is only poorly defined and may vary from protein to protein [15,18]. Linear and conformational epitopes are not equally affected by the acidic and proteolytic environment in the GI tract, where both may have a disruptive effect on the protein structure and thereby on conformational epitopes whereas linear epitopes are only disrupted by proteolysis. Nevertheless, how the environment in the GI tract may impact the types of epitope to which antibodies are raised depends on the structural properties of the given protein. BLG is regarded as a protein resistant to acidic pH as well as proteolytic cleavages in the GI tract, rendering the structure relatively unchanged during passage through the GI system, allowing possible uptake of the protein in its native form, while ALA has been shown to be very susceptible to digestion [15,18]. This correlated very well with our results showing the conformational epitopes of ALA to be more susceptible to the acidic and proteolytic environment of the GI tract than BLG. Structural changes occurring as a result of the environment in the GI tract may generate new conformational epitopes, or expose new antigenic sites on proteins, so-called neo-epitopes, as a consequence of the release of parts of the protein normally buried within the core of the protein and now becoming accessible [47-49]. Even though the amount of linear and conformational epitopes were more or less equal for *β*-casein administration by i.p. or oral route, formation of such neo-epitopes could be the case for this protein as the specific antibody response was shown to be greater for denatured *β*-casein compared to native *β*-casein for some of the BN rats.

The present study shows that food allergens perform very differently; some having an intrinsic capacity to induce antibodies primarily directed against conformational epitopes, like BLG and ALA while others induce a larger amount of antibodies directed against linear epitopes, like *β*-casein. In addition, the impact on type of epitopes to which antibodies are directed are affected differently by the choice of administration route. Such great variation between proteins originating from the same food has also been demonstrated for hen’s egg allergens [39,50] and wheat allergens [43].

## Conclusions

The present study demonstrates the important role of the allergen 3D structure in the development of an IgE-mediated response, stressing that conformational epitopes may have an essential role in food allergy. However, the relative amount of linear versus conformational epitopes varies remarkably between different allergens, showing the great variation in the allergen nature.

## Abbreviations

3D: Three-dimensional; ALA: α-lactalbumin; BLG: β-lactoglobulin; BN: Brown Norway; CD: Circular dichroism; CMA: Cow’s milk allergy; DTT: Dithiothretiol; GI: Gastrointestinal; RT: Room temperature; SMP: Skimmed milk powder; TMB: 3,3’,5,5’-tetramethylbenzidine.

## Competing interests

The authors declare that they have no competing interests.

## Authors’ contributions

JLM participated in the denaturation of proteins, participated in the conduction of the native-gel and performed all ELISAs. JLM performed statistical analyses, contributed to the discussion of the results, wrote a student report on the presented results and reviewed the manuscript. SK contributed to the design and carried out the oral animal study as well as the *β*-casein i.p. animal study. SK reviewed the manuscript. CBM contributed to the design of all animal studies, discussed the results and reviewed the manuscript. IP performed CD spectra, the analyses and graphs of these. IP participated in writing of the manuscript and reviewed the paper. VB designed the denaturation protocol and reviewed the manuscript. KLB drew up the study design. KLB participated in the denaturation of proteins and conduction of native-gel. KLB contributed to the design and carried out the BLG and ALA i.p. animal study. KLB participated in the discussion of the results and converted the student report to a paper manuscript. All authors read and approved the final manuscript.

## Authors’ information

The present work place of Stine Kroghsbo is Unisensor A/S, Allerød, Denmark.
